# *In vivo* study of the GC90/IRIV vaccine for immune response and autoimmunity into a novel humanised transgenic mouse

**DOI:** 10.1038/sj.bjc.6601028

**Published:** 2003-07-01

**Authors:** A Scardino, P Correale, H Firat, M Pellegrini, K Kosmatopoulos, P Opolon, P Alves, R Zurbriggen, R Glück, F A Lemonnier, G Francini, M G Cusi

**Affiliations:** 1 INSERM U. 487, IGR, 39 rue Camille Desmoulins, 94805 Villejuif Cedex, France; 2 Centro di Ricerca Oncofarmacologico, Università di Siena, 53100 Siena, Italy; 3 CNRS URA 1922, Genethon III, 1bis, rue de l'Internationale, 91002 Evry Cedex, France; 4 UMR 1582, IGR, 39 rue Camille Desmoulins, 94805 Villejuif Cedex, France; 5 Virology Department, Berna Biotech, Berne, Switzerland; 6 Immunité Cellulaire Antivirale, Institut Pasteur, 28 rue du Dr Roux, 75724 Paris Cedex 15, France; 7 Dipartimento di Biologia Molecolare, Sezione di Microbiologia, Università di Siena, Via Laterina 8, 53100, Siena, Italy

**Keywords:** antigens, cytotoxicity, tumour immunity, vaccination

## Abstract

Parathyroid hormone-related protein (PTH-rP), a secreted protein produced by prostate carcinoma and other epithelial cancers, is considered a key agent for the development of bone metastases. We investigated the construct GC90/IRIV, composed of immunopotentiating reconstituted influenza virosomes (IRIV) containing PTH-rP gene plasmids (GC90), as a potential tool for human anticancer immunotherapy into humanised mice transgenic for HLA-A(^*^)02.01, the human-*β*2 microglobulin, and the human CD8*α* molecule. Intranasal administration of GC90/IRIV resulted in the induction of a PTH-rP-specific multiepitope cytotoxic T-cell (CTL) response. Cytotoxic T cells derived from vaccinated mice were capable of lysing *in vitro* syngenic murine PTH-rP transfectants and human HLA-A(^*^)02.01^+^/PTH-rP^+^ prostate carcinoma LNCaP cells as well. The immune response capacity and the absence of any sign of toxicity and/or autoimmunity *in vivo* suggest the GC90/IRIV vaccine as a valid tool for active specific immunotherapy of human cancers and metastases overexpressing PTH-rP.

Prostate cancer is the second leading cause of cancer-related death in males in Europe and the US (Zaccagnini, 1999) and the development of hormone-resistant metastatic bone metastases is one of the major causes of morbidity and mortality related to this malignancy. Parathyroid hormone-related protein (PTH-rP) is an attractive candidate as a target antigen in the protocol of active specific immunotherapy of prostate carcinoma and epithelial malignancy bone metastases. It was originally described as the factor responsible for humoral hypercalcaemia of malignancy (HHM) (Suva *et al*, 1987; Francini *et al*, 1993). It is expressed in 90% of prostate and (spindle-cell) lung cell carcinomas and 95% of bone metastases of epithelial cancers, and is produced in small amounts in normal adult tissues (Iguchi *et al*, 1996). It is also responsible for tumour cell growth and survival in the bone tissue, as long as it stimulates osteoclast activity and production of growth factors, cytokines, and TGF-*α*, which in turn promote tumour cell growth and make the bone a feasible microenvironment for tumour growth (Guise, 1997).

Previous studies of our group demonstrated that a specific cytotoxic T-cell (CTL) response *ex vivo* could be elicited from healthy donor PBMC as well as tumour-infiltrating lymphocytes (TILs) derived from prostate carcinoma bone metastases stimulated *in vitro* with autologous dendritic cells (DC) pulsed with peptide epitopes derived from PTH-rP-expressing HLA-A(^*^)02.01-binding amino-acid consensus motifs (Correale *et al*, 2001a; Francini *et al*, 2002). Although some of them expressed nonconventional HLA-A(^*^)02.01-binding motifs, these peptides were all able to induce a PTH-rP-specific CTL response in human PBMCs *in vitro* and in a former HLA-A(^*^)02.01 transgenic mice model *in vivo*.

Different approaches of active immunisation are currently explored for the treatment of human cancer (Fynan *et al*, 1993; Paglia *et al*, 1996; Hwu, 1997) and the role for the DNA-based anticancer vaccine is rapidly rising. In this context, we investigated the capacity of influenza virosomes including PTH-rP gene plasmids to stimulate a specific antitumour CTL response as a delivery system for PTH-rP gene expression. Immunopotentiating reconstituted influenza virosomes (IRIV) are influenza virosomes carrying on the surface the two envelope glycoproteins of the influenza virus, one of which is the influenza haemagglutinin (HA) that facilitates the targeting of plasmid DNA to APCs, improving the transfection efficiency and reducing the rate of DNA degradation by extracellular nucleases (Meyer *et al*, 1995; Dzau *et al*, 1996). We previously showed the GC90/IRIV capacity to elicit a CTL response *in vivo* into BALB/c mice and *in vitro* in human PBMC stimulated with IL-2 and autologous GC90/IRIV-infected DC (Correale *et al*, 2001a).

The aim of this study is to investigate whether GC90/IRIV is able to elicit a CTL response of antitumour efficacy against the PTHrP, by recognising the known HLA-A(^*^)02.01-restricted epitopes, and whether the vaccination with this construct induces significant side effects *in vivo*. The novel humanised mouse model used in this study expresses HLA-A(^*^)02.01 molecules, human *β*2 microglobulin, and human CD8*α* representing an ideal model to investigate vaccines for human utilisation. In fact, murine TCR/human MHC-I-optimised interactions permitted us to study whether the murine CTL response was specific for four HLA-A(^*^)02.01-restricted epitopes directly onto PTH-rP-expressing human tumour cell lines. Furthermore, sequence homology among the human and murine PTHrP required investigation of the CTL response in the same transgenic system as a cause of any undesired side effects or autoimmunity signs *in vivo.*

## MATERIALS AND METHODS

### Triple knockout mice (mouse *β*2 microglobulin, H-2K^b^/D^b^)/triple transgenic for HLA-A(^*^)02.01, human *β*2 microglobulin, and human CD8*α*

Mice triple knockout (KO) (mouse *β*2 microglobulin, H-2K^b^/D^b^)/triple transgenic for HLA-A(^*^)02.01, human *β*2 microglobulin, and human CD8*α* were created at Pasteur Institut. C57BL/6 *β*2 microglobulin-deficient and double mutant H2-K^b^/D^b^ mice have already been described (Pascolo *et al*, 1997; Perarnau *et al*, 1999). These mice were derived by sequential intercrossing of HLA-A(^*^)02.01 transgenic C57BL/6 mice, human *β*2 microglobulin transgenic C57BL/6 mice, human CD8*α* transgenic C57BL/6 mice, mouse *β*2 microglobulin KO mice, and H-2K^b^D^b^ double KO mice. The HLA-A(^*^)02.01 and human *β*2 microglobulin transgenes are in genomic configuration, and the human CD8*α* gene is a cDNA construct under the control of a human CD2 gene promoter. The destruction of the mouse *β*2 microglobulin, mouse H-2K^b^, and D^b^ genes was achieved in embryonic stem cells of 129 origin. Triple transgenic/triple KO mice are homozygote for the six genetic alterations. The genetic background of these mice is largely from C57BL/6 origin with an undetermined proportion of 129 origin. Mice were maintained in our facilities and used for experimentation between 6 and 8 weeks of age. All animal experiments were carried out according to the UKCCCR Guidelines (Workman *et al*, 1998).

### Cell cultures

The EL4/HHD (EL4-S3-Rob/HHD) cells are mouse *β*2 microglobulin-deficient thymoma cells transfected with the HHD monochain construct (Pascolo *et al*, 1997). Transient transfectants EL4/HHD/PTH-rP (EL4-S3-Rob/HHD/PTH-rP) were obtained by transfection of EL4-S3-Rob/HHD cells with GC90 plasmid as previously described (Correale *et al*, 2001b). The prostate carcinoma cell line LNCaP was purchased from the American Type Culture collection (Rockville, MD, USA). Mycoplasma-free cultures of LNCaP and EL4-S3-Rob/HHD were maintained in complete medium (Roswell Park Modified Iscowes (RPMI)-1640, (Life Technologies Inc. (Gibco BRL), Grand Island, NY, USA)) supplemented with 10% foetal bovine serum (FBS), 2 mM L-glutamine, 100 U ml^−1^ penicillin, and 100 *μ*g ml^−1^ streptomycin (Life Technologies, Inc.).

### Peptide synthesis

Peptides PTR-1 (AVSEHQLLH), PTR-2 (FLHHLIAEIH), PTR-3 (WLDSGVTGS), and PTR-4 (TSTTSLELD) were synthesised at the Molecular Biology Department of the University of Siena using a solid-phase automatic peptide synthesiser (model syto, MultiSyntech, Witten, D) and the fluorenylmethoxycarbonyl (Fmoc)/diisopropylcarbodiimide (DIC)/1-hydroxybenzotriazole (HOBT) strategy. They were cleaved from the resins and defracted by treatment with trifluoroacetic acid containing ethanedithiol, water triisopropilsilane and anisole (93 : 2.5 : 2 : 1.5 : 1). The crude peptides were purified by high-performance liquid chromatography (HPLC) using a Vydac C18 column (25 cm × 1 cm, 10 *μ*m). The products were dissolved in double-distilled water, sterile filtered, and frozen at −70°C at a concentration of 2 mg ml^−1^. Peptide purity was more than 90% as analysed by HPLC. The CAP-1 peptide was kindly provided by Dr J Schlom (EOS, NCI, Bethesda, MD, USA).

### ELISpot assay

Peripheral lymphocytes isolated from blood samples collected from the retro-orbitary sinus of the different groups of mice were pooled and examined for PTH-rP epitope peptide-specific precursor frequency by using the interferon-*γ* (INF-*γ*) ELISpot assay (Miyahira *et al*, 1995). Briefly, nitrocellulose-bottomed 96-well plates (Millipore, Billerica, MA, USA) were coated for 2 h at 37°C followed by overnight incubation at 4°C with rat anti-mouse IFN-*γ* antibody (clone R4-6A2; Pharmingen). Dilutions of responder cells in complete medium were cultured in triplicate with or without 10 *μ*M peptide epitope for 40 h. Plates were then washed and incubated with biotinylated IFN-*γ* antibody (clone XMG1.2; Pharmingen, Heidelberg, Germany) followed by the streptavidin–alkaline phosphatase conjugate (Roche Diagnostics, Indianapolis, IN, USA). Spots were visualised using BCIP/NBT alkaline phosphatase substrate (Promega, Madison, WI, USA). Interferon-*γ*-secreting cells were counted using the automated image analysis system ELISpot Reader (AID Strassberg, Germany). The Wilcoxon two-tail-rank test was performed to determine whether there was a statistically significant difference between the number of IFN-*γ*-secreting cells in the wells stimulated with or without the peptides of interest or with an aspecific peptide (HIVgag76 peptide).

### Generation of a PTH-rP plasmid and influenza virosomes

The PTH-rP gene was amplified from the DU-145 prostate carcinoma cell line by means of reverse transcriptase–polymerase chain reaction (RT–PCR) (Correale *et al*, 2001b) starting from the specific mRNA by using the sense primer 5′TTGGATCCATGCAGCGGAGACTGGTT3′ and the antisense primer 5′CCGAATT CTCAATGCCTCCGTGAATCGA3′, and cloned in *Bam*HI-*Eco*RI sites of the pcDNA3 expression vector (InVitrogen, Karlsruhe, Germany) in order to obtain the recombinant plasmid GC90. The construct was grown in DH5*α* cells (Life Technologies Inc.). Plasmid DNA was purified using the Qiagen Endo Free plasmid kit (QIAGEN, Hilden, Germany) as described by the manufacturer. The influenza virosomes (IRIV) were prepared as described elsewhere (Wâelti and Glück, 1998). Nonencapsulated plasmids were separated by 0.1 gel filtration on a High Load Superdex 200 column (Amersham Pharmecia Biotech Uppsale, Sweden) equilibrated with sterile phosphate-buffered solution (PBS). The void volume fractions containing the virosomes and encapsulated plasmids were eluted with PBS and collected.

### Cell transfection

A total of 1.0 × 10^6^ target cells were grown in six-well microplates at 37°C and infected with 0.3 *μ*g of DNA-virosomes or transfected with 1 *μ*g of plasmid DNA using the Effectene Transfection reagent (QIAGEN) as described by the manufacturer. After 2 days, PTH-rP antigen expression was analysed by evaluating the presence of the specific mRNA by RT–PCR and by immunofluorescence. Briefly, the cells were washed twice with PBS, fixed with cold methanol/acetone, and treated with a rabbit anti-PTH-rP serum (Calbiochem, SanDiego, USA) followed by FITC-conjugated goat anti-rabbit IgG (1 : 100) (DBA, DBA s.r.l. Milan, Italy). The slides were examined using a Diaplan microscope (Leitz, Oberkochen, Germany).

### Immunisation of triple KO/triple transgenic mice

Five groups of six triple KO/triple transgenic mice received 20 *μ*l of GC90/IRIV (containing 5 *μ*g of plasmid, 0.6 *μ*g of influenza HA, and 40 ng of Escheriagen, *Escherichia coli* heat-labile toxin) after intranasal (i.n.) inoculation. Mice in the control groups received i.n. inoculation of 20 *μ*l of IRIV, or 20 *μ*l IRIV containing the plasmid backbone (pcDNA3). All groups of mice, with the exception of those included in the control groups, were subsequently reboosted 21 and 42 days after the first immunisation with GC90/IRIV; PTR-1, -2, -3, and -4 respectively. Parathyroid hormone-related protein peptides were administered by subcutaneous (s.c.) injection at the base of the tail with 100 *μ*g of peptide emulsified in incomplete Freund's adjuvant (IFA) in the presence of 140 *μ*g of the IA^b^ restricted HBVcore-derived T-helper epitope (128–140; sequence TPPAYRPPNAPIL). At 21 and 56 days after the first immunisation, sera samples were collected from the retro-orbital sinus for serum Ca^2+^ ion level evaluation. At 2 weeks after the final boost, the mice were killed and 4 *μ*M-thick paraffin sections were made from sampled tissues and stained with haematoxylin–eosin–safranin (Merck, Germany) (Workman *et al*, 1998). Spleen cells (5 × 10^7^ cells in 10 ml) were harvested on day 56 and cultured for 6 days in *serum-free* AIM-V (Life Technologies Inc. (Gibco BRL)), with 2 mM L-glutamine, 100 U ml^−1^ penicillin, 100 *μ*g ml^−1^ streptomycin, and 100 IU of interleukin 2, and *in vitro* stimulated with autologous irradiated spleen cells transfected with GC90 plasmid +/− the cognate peptide (10 *μ*M) used for mouse reboosting. After 6 days, the bulk responder populations were tested for PTH-rP-specific cytotoxicity.

### Cytotoxicity assay

Target cells were labelled with 100 *μ*Ci of Na_2_Cr^51^O_4_ (Amersham, Aylesbury,UK) for 60 min at room temperature. Target cells (0.5 × 10^4^) in 100 *μ*l of complete medium (see below) were added to each of the wells in 96-well flat-bottomed assay plates (Corning Costar Corp., Cambridge, MA, USA). The labelled targets were incubated at 37°C in 5% CO_2_ before the addition of effector cells. The T cells were then suspended in 100 *μ*l of AIM-V medium and added to the target cells. The plates were incubated at 37°C for 6 h, and the supernatants harvested for *γ*-counting with harvester frames (Skatron, Inc., Sterling, VA, USA). The determinations were made in triplicate and standard deviations were calculated. All of the experiments were repeated at least three times.

Specific lysis was calculated as follows:


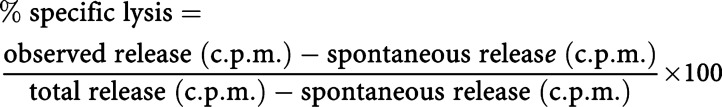


Spontaneous release was determined from the wells to which 100 *μ*l of complete medium was added instead of effector cells. Total releasable radioactivity was obtained after treating the target with 2.5% Triton X-100.

### Blocking experiments

For HLA-blocking experiments, UPC-10 (Cappel/Organon Technique Corp., West Chester, PA, USA) control mAb or anti-HLA-A2 (A2.69, #189HA-1; One Lambda, Inc., Canoga Park, CA, USA) mAb were added to the ^51^[Cr] loaded target cells (EL4/HHD/PTH-rP and LNCaP) and incubated for 1 h prior to the cytotoxic assay.

### Statistical analysis

Statistical analysis of differences between means was performed using Stat View statistical software (Abacus Concepts, Berkeley, CA, USA). The results were expressed as the mean of four determinations derived from two different experiments±standard deviation. Differences among means were determined by the two-tailed Student's *t*-test for paired samples.

## RESULTS

### Vaccination of transgenic mice with GC90/IRIV

In order to evaluate its immunogenic potential, we administered GC90/IRIV intranasally to five different groups of triple transgenic mice. After 21 days, IFN-*γ* ELISpot assays for the four HLA-A(^*^)02.01 peptides PTR-1, -2, -3, and -4, were carried out on pooled peripheral lymphocytes of all mice groups. As shown in Table 1

**Table 1:** Peptide-specific CTL freshly isolated from peripheral cells of mice^a^ INF-*γ* secreting cells/10^6^ peripheral cells

**Vaccination protocol**		**PTH-rP peptides (1 *μ*g ml^−1^)**			
**Priming**	**Reboost**	**PTR-1**	**PTR-2**	**PTR-3**	**PTR-4**
GC90/IRIV^b^	No	18±3*	15±1*	29±3*	13±1*
IRIV^b^	No	4±1***	5±1***	5±2***	4±1***
GC90/IRIV	GC90/IRIV^c^	50±2*	70±7*	48±2*	32±4*
GC90/IRIV	PTR-1^c^	16±2**			
GC90/IRIV	PTR-2^c^		33±3*		
GC90/IRIV	PTR-3^c^			12±2**	
GC90/IRIV	PTR-4^c^				5±1**
IRIV	IRIV^c^	4±2***	8±2***	10±3***	3±1***

CTL=Cytotoxic T-cells; INF-*γ*=interferon-*γ*; PTH-rP=parathyroid hormone-related protein.

* *P*<0.01 compared with the respective intra-assay controls.

** *P*=0.05–0.01 compared with the respective intra-assay controls.

*** *P*>0.05 compared with the respective intra-assay controls.

a Cytotoxic T-cell response was evaluated *ex vivo* on pooled peripheral cells taken from the retro-orbital sinus. Specific spot numbers were obtained by subtraction of the background. Results represent the mean of wells in triplicate.

b At 21 days from the first immunisation.

c At 56 days from the first immunisation.

, a multiepitope PTH-rP peptide-specific response was observed after a single inoculation of GC90/IRIV (Table 1). In order to investigate the efficacy of reboost with PTH-rP peptides, the PTH-rP-specific CTL response was then tested in the five separate groups of GC90/IRIV-vaccinated mice that had received a reboost with GC90/IRIV or a peptide. The first group was reboosted with GC90/IRIV, while the other four groups were reboosted with each one of the four PTH-rP peptides. Peripheral lymphocytes derived from all the groups were collected 56 days after the priming and examined by IFN-*γ* ELISpot assays for their capacity to recognise the four PTH-rP peptides. Results show a multiepitope-specific CTL response in mice receiving a reboost with GC90/IRIV, while a lower number of cognate peptide-specific T-cell precursors frequency was detected in mice reboosted with the single PTH-rP peptides (Table 1). The two additional groups used as a negative control showed no response at all. Taken together, these results suggest that GC90/IRIV is immunogenic *in vivo* and that reboosting with the same construction is more effective than with PTH-rP peptide alone to augment the number of specific precursors *in vivo*.

### Parathyroid hormone-related protein- specific antitumour activity of CTL derived from GC90/IRIV-vaccinated mice

**Figure 1:**
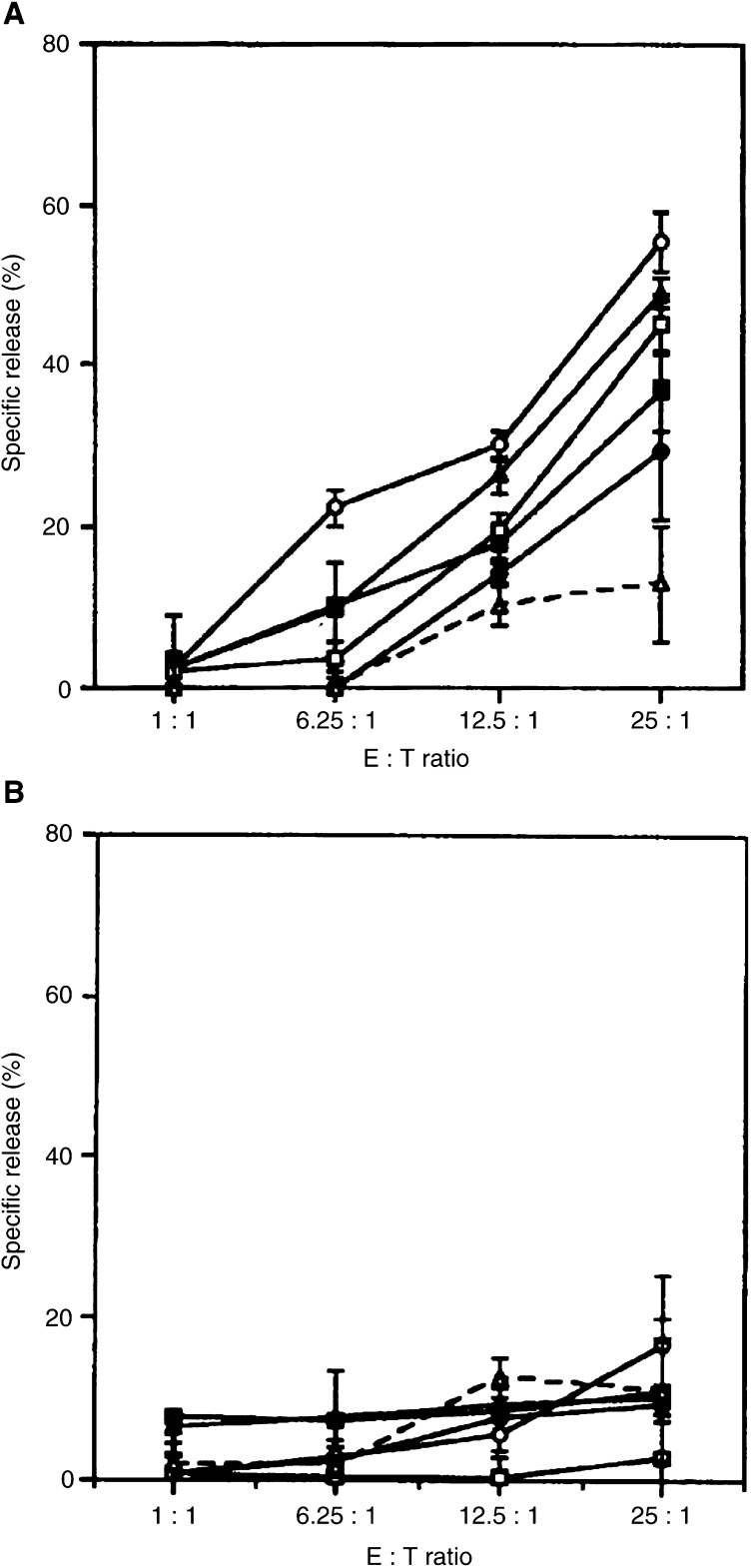
Parathyroid hormone-related protein-specific cytotoxic activity of spleen cells derived from triple transgenic mice immunised with GC90/IRIV +/− PTH-rP peptides. Cultured spleen cells derived from different groups of mice respectively immunised with GC90/IRIV (-•-), GC90/IRIV+PTR-1 (-▪-), GC90/IRIV+PTR-2 (-▴-), GC90/IRIV+PTR-3 (-○-), GC90/IRIV+PTR-4 (-□-), and empty IRIV group (–▵–). Parathyroid hormone-related protein-specific cytotoxic activity of mouse spleens pooled from different mouse groups was tested against EL4/HHD target cells transfected with the PTH-rP gene (**A**) in fresh medium or in the presence of anti-A2.69 mAb (**B**).

**Figure 2:**
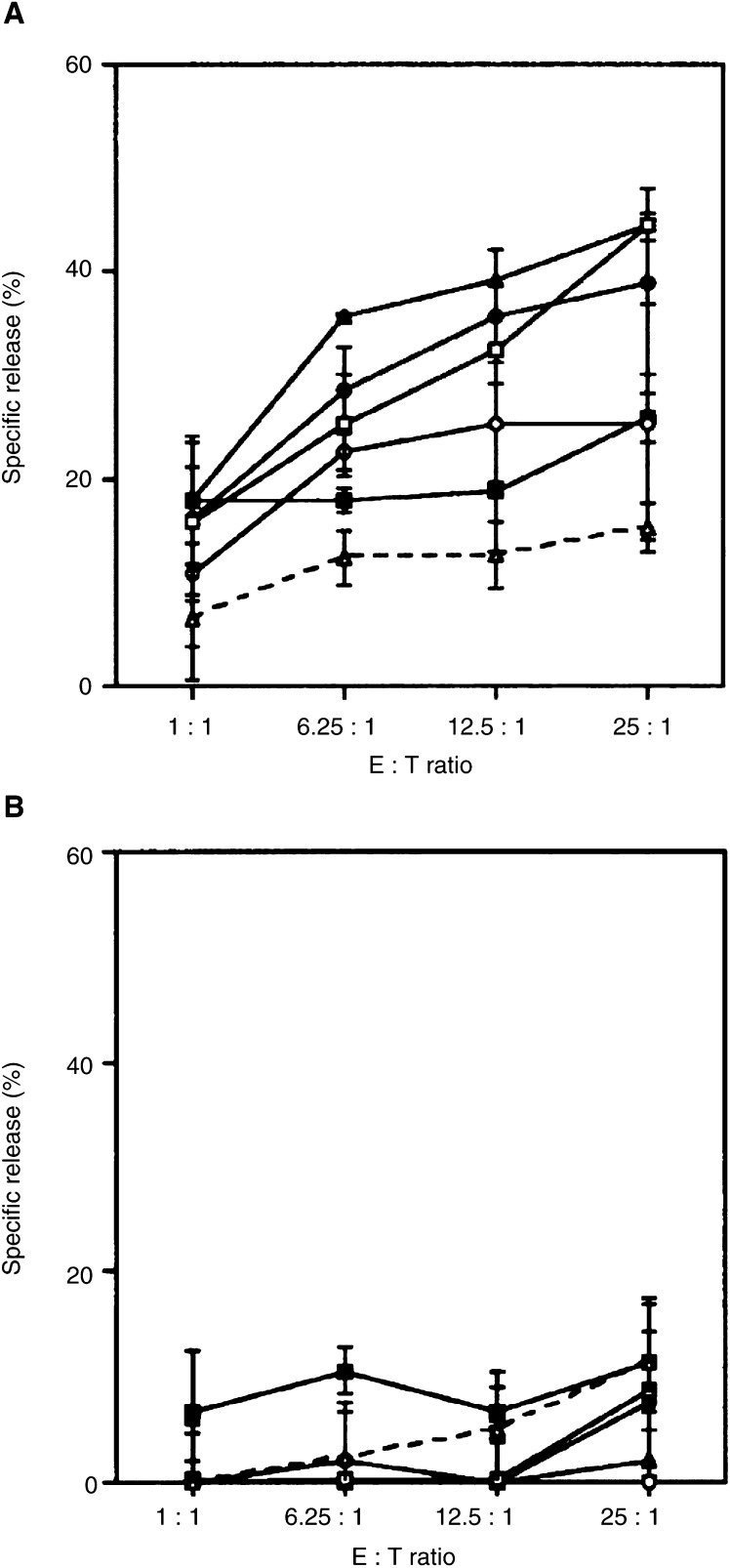
Parathyroid hormone-related protein-specific cytotoxic activity of spleen cells derived from triple transgenic mice immunised with GC90/IRIV +/− PTH-rP peptides *in vitro* against HLA-A(^*^)02.01^+^ PTH-rP^+^ LNCaP cells. Cultured spleen cells were derived from different groups of transgenic mice respectively immunised with GC90/IRIV (-•-), GC90/IRIV+PTR-1 (-▪-), GC90/IRIV+PTR-2 (-▴-), GC90/IRIV+PTR-3 (-○-), GC90/IRIV+PTR-4 (-□-), and empty IRIV group (–▵–). Parathyroid hormone-related protein-specific cytotoxic activity of mouse spleens pooled from different mouse groups was tested against LNCaP target cells in fresh medium (**A**) or in the presence of anti-A2.69 mAb (**B**).

The PTH-rP-specific cytotoxicity of the CTL response was investigated in a 6 h ^51^Cr release assay. Spleen cells derived from the different groups of mice vaccinated with GC90/IRIV and reboosted with GC90/IRIV or with each one of the PTH-rP peptides were collected and tested for their capacity to kill HLA-A(^*^)02.01^+^ target cells expressing PTH-rP. Controls were represented by splenocytes derived from mice vaccinated with the empty IRIV/pcDNA3 alone. The spleen cells derived from the different immunisation groups were stimulated *in vitro* with low-dose IL-2 and autologous irradiated spleen cells induced to express PTH-rP protein after transfection with GC90 plasmid. CTL cultures derived from mice vaccinated with GC90/IRIV were able to kill murine EL4/HHD/PTH-rP target cell transfectants (Figure 1

) as well as the human HLA-A(^*^)02.01^+^/PTH-rP^+^ prostate carcinoma cell line LNCaP (Figure 2

). Cytotoxic T-cell cultures derived from mice primed with GC90/IRIV and boosted with each of the four PTH-rP peptides also showed a lytic capacity against both the targets (Figures 1 and 2), though with a lower extent for the CTL cultures derived from mice reboosted with PTR-1 peptide (Figures 1 and 2). Cytotoxic T-cell cultures generated from not vaccinated control mice or from mice vaccinated with IRIV/pcDNA3, but still *in vitro* stimulated with GC90 plasmid transfected spleen cells, showed weak cytotoxic activity against the EL4/HHD/PTH-rP transfectants (Figure 1), and were not able to lyse the LNCaP target cell line (Figure 2). None of the CTL cultures was able to kill murine EL4/HHD target cell lines infected with the empty pcDNA3/IRIV vaccine as a control (Figure 1). The lysis of murine EL4/HHD/PTH-rP transfectants and human LNCaP target cells was HLA-A(^*^)02.01 restricted since it was completely abrogated by an anti-HLA-A(^*^)02.01 mAb (A2.69) (Figures 1B and 2B). Conversely, the use of a negative control mAb did not affect the target cell killing (data not shown). These results demonstrate that vaccination of triple transgenic mice with GC90/IRIV generated *in vivo* a PTH-rP-specific CTL response able to kill tumour targets naturally processing the PTH-rP molecule.

### *In vivo* study after vaccination of triple transgenic mice with GC90/IRIV

The sequence homology between the human and murine PTH-rP protein sequences is >90%. Amino-acid homology between the human PTR peptides and sequences of corresponding murine PTH-rP peptides was 100% for PTR-1 and PTR-2, and 60% for PTR-3 and PTR-4. Tissue-specific toxicity and autoimmunity induced by GC90/IRIV vaccination was then evaluated *in vivo* into transgenic mice. All vaccinated animals were killed at day 56 after the first immunisation and analysed by histology of tissues selected for PTH expression such as parathyroids, and PTH-rP for skin, derma, and breast. Histology samples showed no signs of pathologic microscopic lymphocyte infiltration of selected tissues or any abnormal inflammation status (data not shown). These results suggest that the GC90/IRIV vaccination generates a CTL response specific for PTH-rP that is not able to affect the normal tissues *in vivo*. Considering that the transient expression of the whole PTH-rP protein in mice vaccinated with GC90/IRIV could affect their physiologic Ca^2+^ turnover, serum levels of Ca^2+^ ions were monitored during treatment in all mice groups (blood samples collected 21 and 56 days after the first inoculation of GC90/IRIV). There was no evidence of important serum Ca^2+^ ion fluctuations in the vaccinated group (Table 2

**Table 2:** Serum [Ca^2+^] levels in mice after vaccination with GC90/IRIV±PTH-rP peptides

**Vaccination protocol**	**Means of three mice**	
	**Day 21**	**Day 56**
Thyrocalcitonin^a^	0.55±0.02	
No vaccine	1.32±0.1	1.57±0.03
IRIV-IRIV	1.07±0.1	1.45±0.005
GC90/IRIV–GC90/IRIV	1.19±0.1	0.89±0.2
GC90/IRIV–PTR-1	1.18±0.2	1.09±0.2
GC90/IRIV–PTR-2	1.06±0.1	1.00±0.2
GC90/IRIV–PTR-3	1.16±0.2	1.10±0.1
GC90/IRIV–PTR-4	1.10±0.2	0.96±0.1

a The positive control was represented by a group of three mice injected s.c. in the dorsal neck with human synthetic thyrocalcitonin (0.1 mg in 1 ml of 0.9% saline solution) inducing significant hypocalcaemia values after 6 h. Values of Ca^2+^ concentration are given as mmol l^−1^±s.d.

) by comparison to a positive control group of three mice injected s.c. in the dorsal neck with human synthetic thyrocalcitonin (0.1 mg in 1 ml of 0.9% saline solution) showing a significant hypocalcaemia after 6 h (0.55 of mean, *P*<0.03). These results indicate that GC90/IRIV is able to elicit a PTH-rP-specific CTL response in transgenic animals without pathologic effect on the bone osteoclastic activity or metabolism of Ca^2+^ *in vivo*.

## DISCUSSION

In the present study we evaluated the immunological activity of GC90/IRIV vaccine into a novel humanised mouse model triple KO (mouse *β*2 microglobulin, H-2K^b^/D^b^)/triple transgenic (HLA-A(^*^)02.01, human *β*2 microglobulin, human CD8*α*). This mouse represents a warrant for the possibility to test immune response to antigen-derived epitopes with amino-acid anchor motifs specific for the human HLA-A(^*^)02.01 molecule. This mouse strain also expresses the human CD8*α* in the form of homodimers on the T-cell membrane for optimal interactions with the *α*3 domain of the HLA-A(^*^)02.01 molecule. In fact, several experiments, including crystallographic study, have documented the role of such molecules for correct interactions with human class I HLA molecules (Wesley *et al*, 1993; Sun *et al*, 1995; Gao *et al*, 1997). In this model, such interaction allows murine CTLs to lyse tumour cells of human origin. In addition, the absence of murine class I H-2K^b^ and H-2D^b^ in these animals permits an efficient education of HLA-A(^*^)02.01-specific CTL in the thymus as well as in the periphery (Tanchot *et al*, 1997). In fact, the expression of the human CD8 molecule on murine CTL engenders an optimal TCR/peptide/MHC trimolecular complex interaction, especially onto human target cells, allowing the direct evaluation of the immune response of anticancer vaccines for human purposes (Germain, 1994). Furthermore, the possibility to generate an immune response against tumour-associated antigen (TAA) expressed by human malignancies in such an animal model is coupled with the opportunity to monitor the occurrence of undesirable side effects and autoimmunity phenomena.

In this model, we found that mucosal infection with the GC90/IRIV was able to elicit a significant multiepitopic PTH-rP-specific CTL response with antitumour activity. This observation was of particular interest as long as CTL precursors, stimulated by vaccination and reboosted with GC90/IRIV, were able to recognise the known HLA-A(^*^)02.01 PTH-rP epitopes (PTR-1, -2, -3, and -4) expressed in human cancer cells. In the present study, we also provide evidence that vaccination and reboost of transgenic mice with the same GC90/IRIV is able to elicit a stronger multiepitopic CTL response specific for PTH-rP when compared with a reboost approach designed with synthetic peptides only. A possible explanation could be the fact that the APC engagement and help signals provided by the mucosal inoculation route of GC90/IRIV construction are more effective in CD8+ response promoting than s.c. peptide injection in IFA. In fact, previous studies revealed that antigen encapsulated in liposomes could be successfully delivered simultaneously into the cytosolic as well as endosomal processing pathways of APCs, leading to the generation of both CD4+ T helper and CD8+ cytotoxic T cells (Owais *et al*, 2001). It might be useful to further investigate such aspects in order to highlight and potentiate GC90/IRIV-mediated mechanisms of CTL priming.

In this study, as an unexpected result we observed that the sequential administration of PTH-rP epitope peptide after the primary vaccination with GC90/IRIV determined a selective reduction in the frequency of CTL precursor specific for the cognate PTR peptide injected for re-stimulation. Three major hypotheses were formulated in order to explain such a finding: (1) administered peptides entering the blood circulation bind to empty HLA-A^*^02.01^+^ molecules on normal cells and may determine the specific epitope peptide precursors' lineage anergy and deletion by HLA-A^*^02.01/peptide complex interaction with the specific TCR in the absence of the correct co-accessory molecule interaction (Perarnau *et al*, 1999); (2) at least three GC90/IRIV administrations are needed to induce an effective PTH-rP-specific immune response suggesting that the GC90/IRIV construct provides a better helper response for CTL generation than peptides injected with IFA adjuvant; (3) peptide administration may determine the activation of an epitope peptide-specific CTL population that migrates into the injection site draining lymph nodes or derma. Our results seem to support the last hypothesis because a stronger PTH-rP-specific cytolytic response to prostate carcinoma LNCaP cells was observed in CTL lines derived from spleen cells taken from mice receiving the sequential GC90/IRIV/peptide treatment.

Furthermore, peptides used for this study and derived from the human PTH-rP sequence were chosen for their sequence homology with the murine sequence of the same protein. Interestingly, the PTH-rP-specific CTL response obtained after vaccination could not engender any autoimmunity sign *in vivo* observed by the histology of organs from vaccinated mice, excluding the caveat to the cellular response against self tissues after vaccination with this construct. This last evidence is probably due to the quality of the CTL repertoire recruited and more likely to the low level of PTH-rP expression *in vivo* in normal tissues. In fact, the endogenous PTH-rP epitope peptide levels could be too poor to be detected by the CTLs that are conversely able to recognise *in vitro* the same antigen overexpressed on tumour cells.

Weak Ca^2+^ ions reduction is present in the group of mice immunised with GC90/IRIV, revealing mild hypocalcaemia. GC90/IRIV vector does not let the DNA of PTHrP protein to be integrated into the host DNA, as it happens for lentiviral or retroviral vectors. Thus, one hypothesis is that the transient Ca^2+^ fluctuation may be due to the temporary presence of circulating PTH-rP and this should rapidly return to normal levels with the circulating protein disappearance. In fact, calcium ion levels are strictly under the control of the parathyroid hormone (PTH), which is produced by parathyroids, and shows amino-acidic homologies with PTH-rP. Another hypothesis is that mice vaccinated and reboosted with GC90/IRIV may produce PTH-blocking antibodies and may determine alteration in PTH-producing cells. Further experiments are presently in course to investigate this hypothesis.

In conclusion, this study presents GC90/IRIV as a good vaccine candidate to be investigated in clinical trials for human cancers and bone metastases overexpressing PTH-rP. In addition, this is the first description of the triple KO/triple transgenic mice that appears to be a versatile model employed for preclinical studies of cancer vaccines for the human HLA-A(^*^)02.01 haplotype background.
